# ZBMG-LoRa: A Novel Zone-Based Multi-Gateway Approach Towards Scalable LoRaWANs for Internet of Things

**DOI:** 10.3390/s25175457

**Published:** 2025-09-03

**Authors:** Mukarram Almuhaya, Tawfik Al-Hadhrami, David J. Brown, Sultan Noman Qasem

**Affiliations:** 1 Computer Science Department, School of Science and Technology, Nottingham Trent University, Clifton, Nottingham NG11 8NS, UK; nmnmzair@gmail.com (M.A.); tawfik.al-hadhrami@ntu.ac.uk (T.A.-H.); david.brown@ntu.ac.uk (D.J.B.); 2 Computer Science Department, College of Computer and Information Sciences, Imam Mohammad Ibn Saud Islamic University (IMSIU), Riyadh 11432, Saudi Arabia

**Keywords:** Internet of Things, LPWAN, LoRa, LoRaWAN, adaptive data rate

## Abstract

Internet of Things (IoT) applications are rapidly adopting low-power wide-area network (LPWAN) technology due to its ability to provide broad coverage for a range of battery-powered devices. LoRaWAN has become the most widely used LPWAN solution due to its physical layer (PHY) design and regulatory advantages. Because LoRaWAN has a broad communication range, the coverage of the gateways might overlap. In LoRa technology, packets can be received concurrently by multiple gateways. Subsequently, the network server selects the packet with the highest receiver strength signal indicator (RSSI). However, this method can lead to the exhaustion of channel availability on the gateways. The optimisation of configuration parameters to reduce collisions and enhance network throughput in multi-gateway LoRaWAN remains an unresolved challenge. This paper introduces a novel low-complexity model for ZBMG-LoRa, mitigates the collisions using channel utilisiation, and categorises nodes into distinct groups based on their respective gateways. This categorisation allows for the implementation of optimal settings for each node’s subzone, thereby facilitating effective communication and addressing the identified issue. By deriving key performance metrics (e.g., network throughput, energy efficiency, and probability of effective delivery) from configuration parameters and network size, communication reliability is maintained. Optimal configurations for transmission power and spreading factor are derived by our method for all nodes in LoRaWAN networks with multiple gateways. In comparison to adaptive data rate (ADR) and other related state-of-the-art algorithms, the findings demonstrate that the novel approach achieves higher packet delivery ratio and better energy efficiency.

## 1. Introduction

**Figure 1:**
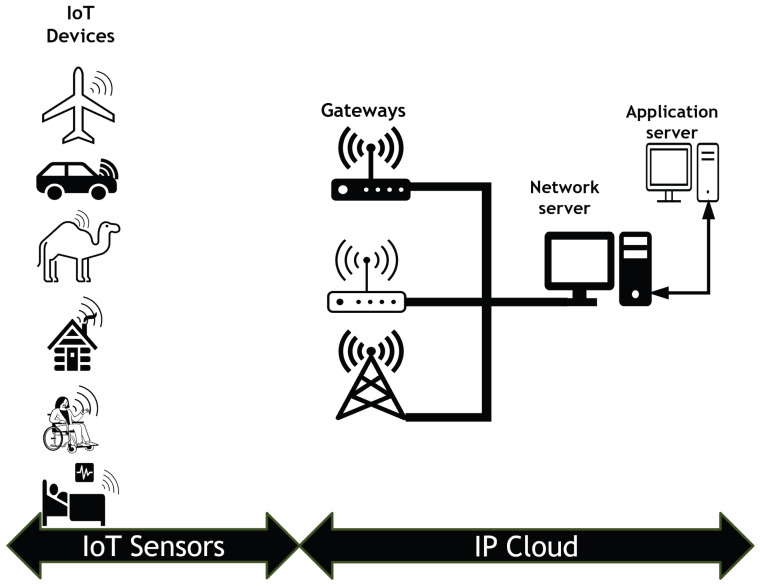
LoRa network architecture.

The Internet of Things (IoT) provides innovative solutions to complex challenges across various sectors, including healthcare, agriculture, and transportation. Organisations can enhance development, innovation, and competitiveness in an interconnected environment through the utilisation of IoT. The Internet of Things is reshaping our lifestyles, professional environments, and technological interactions, fostering a future characterised by enhanced efficiency, connectivity, and data utilisation [1]. One of the challenges currently faced in the IoT field is the provision of assistance to applications requiring a large quantity of inexpensive and durable battery-operated devices capable of communicating over large distances. The IoT landscape is significantly impacted by the significance of LoRa (long range) technology in the domain of low-power wide-area networks (LPWANs). LoRa technology is recognised for its minimal power consumption and long-range communication capabilities, which are instrumental in facilitating cost-effective and efficient connectivity for IoT devices over extended distances [2]. Unlike the common direct sequence spread spectrum system [3], LoRa is a Chirp Spread Spectrum modulation technology [4] and fair frame time slot LoRaWAN [5,6]. The LoRaWAN architecture, seen in Figure 1, is a star topology that comprises nodes, an application server, a network server (NS), and gateways (*GWs*). The nodes send packets to the *GWs* via uplink communication, and the *GWs* send them on to the NS [7]. It is crucial to acknowledge that LoRaWAN transmission performance cannot be guaranteed, despite the implementation of simultaneous packet decoding. The rationale is derived from the random access technique used in LoRaWAN [8]. More precisely, in LoRaWAN, the Aloha-type random access mechanism allows each node to decide when to access the channel, which leads to collision and increases the unsuccessful reception rate. The Aloha protocol is renowned for its ease of implementation and simplicity, but it is plagued by poor performance due to the potential for a large number of channel access requests to occur simultaneously [9]. This may result in numerous packet collisions, even when several base stations are working together to receive them. Developing an effective strategy to manage the increasing number of IoT devices and applications supported by LoRaWAN is crucial for the successful implementation of LoRaWAN in large-scale wide-area IoT scenarios with massive machine-type communications (mMTCs). The existing literature discussing multi-cell Aloha networks with individual packet decoding has consistently noted that the configuration parameters—including the channel access probability—have a substantial impact on throughput performance [10]. Multiple gateways could be a solution in a LoRa network because the packet can be received by numerous gateways, and the network server subsequently selects the packet with the strongest signal [11], thereby improving reliability [12]. Nevertheless, the packet overlaps between multiple gateways when it is sent to more than one simultaneously. Furthermore, this behaviour depletes the availability of channels on gateways. Essentially, a packet can be decoded successfully if it is received by one gateway (at least) with perfect settings. Optimal performance in multi-GW LoRaWAN with joint packet decoding requires proper configuration parameters. Nevertheless, the ideal adjustment of configuration parameters in LoRaWAN is still uncertain.

This paper asserts a two-step algorithm to significantly improve the reliability and scalability of multi-gatway LoRaWAN networks, with the main contributions as follows:

- First contribution: Stopping the reception of packets in all gateways simultaneously prevents wasting channel availability, enabling the network server to allocate other nodes to free channels and assign them to the most suitable gateway.
- Second contribution: The paper formulates a multi-objective optimisation problem to determine the optimal ratio of nodes at each gateway and *SF* zone. This aims to substantially improve LoRaWAN network throughput while minimising energy consumption.
- Third contribution: Nodes are allocated to the nearest zone to meet the target ratio in a multi-gateway setup, then to the next zone, thus avoiding channel wastage and ensuring efficient configurations.

This method involves thoroughly evaluating all paths between the end device and the gateways in the coverage area. It is essential to assess the link budget and select the path with the lowest path loss to ensure the best path and gateway are chosen, thus guaranteeing a highly reliable connection. Our suggested technique concurrently configures the *GWs*, *SFs*, *TPs*, and *Chs* for all nodes in LoRaWAN networks with gateways and spreading factor zones compared to the state-of-the-art. Furthermore, it circumvents the use of SF11 and SF12, which are the primary factors contributing to the rise in collisions. Unlike the work in [13], it employs SF11 and SF12. That is why our approach outperforms other known approaches in terms of packet delivery ratio, throughput, and overall energy usage. Furthermore, it does not need any synchronisation procedure and may be used for class A with an unverified transmission mode. In this mode, nodes do not seek an acknowledgement from the network server after each transfer. In contrast to the works in [14,15,16,17,18,19,20], our proposed algorithm is compared with ADR and other state-of-the-art algorithms. The findings demonstrate that our solution significantly enhances the network energy efficiency for the same reasons mentioned previously. This article is structured into sections in the following manner: Section 2 provides a background and related previous work in this field. The system model is introduced in Section 3. The method ZBMG-LoRa, which we propose, is presented in Section 4. Section 5 focuses on network scenarios and analyses outcomes of the proposed algorithm in comparison to ADR and other advanced algorithms. This paper is concluded in Section 6, where we also propose our future efforts.

## 2. Related Works

Extensive research has been conducted on LoRaWAN, beginning with the single-GW scenario. The study in [21] offered empirical findings that demonstrated the impact of several system characteristics, including spreading factor, payload size, coding rate (CR), and packet transmission interval, on LoRaWAN performance. In [22], decentralised dynamic *SF* allocation algorithms were presented, using deep reinforcement learning to minimise energy usage and enhance network throughput. Based on stochastic geometry, it was demonstrated that LoRaWAN performance decreases dramatically as the number of nodes increases [16,23]. This suggests that in densely distributed networks, the network performance is limited by interference among nodes rather than noise. According to the findings in [24], the packet error rate in LoRaWAN increases as the network load increases. The study also determined the maximum network load that guarantees reliable communication. In another study, [25], the performance of LoRaWAN was examined. It was discovered that to meet a certain quality of service (QoS) criterion in a LoRaWAN with a single gateway, the number of devices must be restricted. In order to enhance multi-GW LoRaWAN performance, *SF* allocation algorithms were offered for the multi-GW situation [26,27]. In [26], a technique called spreading factor with priority (SF-P) was introduced to achieve an optimum distribution of nodes in a multi-GW LoRaWAN. This approach aims to enhance the network performance and accommodate various IoT applications while also considering the specific priority needs of each application. To improve the packet delivery ratio in multi-GW and single-GW LoRaWAN networks as new devices are added, an *SF* allocation algorithm was suggested in [27].

The allocation strategy relies on the link PDR network throughput and the distribution of spreading factors per gateway in the network. Other studies, as in [28,29], utilised simulators to examine the multi-GW LoRaWAN performance under various conditions. In [28], the authors provide a detailed analysis of multi-GW LoRaWAN performance in the EU868 MHz spectrum and the 2.4 GHz spectrum using a simulator. In dense scenarios with a variable number of gateways and bidirectional network traffic, the adaptive strategies performance, specifically the adaptive data payload (ADP) and the adaptive data rate, is compared in [29]. In [30], the authors developed an algorithm that can accurately determine the number of gateways that need to be activated based on the downlink traffic demand in a network. This algorithm is particularly useful in situations where the positions of the nodes are not known. In [31], efforts focused on enhancing the efficiency of multi-GW systems by considering the aspect of diversity. In [11], a novel cooperative decoding scheme was presented, which utilises *GW* diversity to enhance decoding reliability. This scheme takes advantage of multiple copies of the same packet received by different gateways. In [31], it was suggested to incorporate spatial diversity into a multi-GW LoRaWAN network. This approach assists in reducing the impact of path attenuation and enables the successful decoding of weak signals that would otherwise be undetectable. Researchers in [32] introduced a capture-based model that aims to optimise inter-packet error correction codes (ECCs) to ensure dependable communication. In [33,34], stochastic geometry methodologies were employed to evaluate multi-GW LoRaWAN efficiency. The researchers made the assumption that the spatial distribution of nodes adheres to a Poisson point process. The majority of previous studies aimed at enhancing the performance of LoRaWAN have focused on resource allocation, specifically the spreading factor [22,27]. Among LoRaWAN’s random access schemes, the Aloha-type has received little attention. The throughput performance is significantly affected by the setup parameters, such as the channel access probability, according to the previous research on multi-cell Aloha networks with individual packet decoding [10]. The correct setup settings are essential for the multi-GW LoRaWAN with cooperative packet decoding to function well. Nevertheless, the ideal adjustment of configuration parameters in LoRaWAN is still uncertain. This paper presents a two-step algorithm designed to enhance the reliability and scalability of LoRaWAN networks utilising multiple gateways. Firstly, this method involves thoroughly evaluating all paths between the end device and the gateways in the coverage area. It is essential to assess the link budget and select the path with the lowest path loss to ensure the best path and gateway are chosen, thus guaranteeing a highly reliable connection. Secondly, we have formulated a multi-objective optimisation problem to definitively determine the optimal ratio of nodes at each gateway and each *SF* zone. This is aimed at significantly enhancing the LoRaWAN network throughput while unequivocally minimising energy consumption.

### 2.1. LoRa/LoRaWAN

The LoRa system offers significant processing advantages for improved link budgets and resilience to multipath and interference. It is a patented technique of Chirp Spread Spectrum (CSS) modulation that incorporates a Forward Error Correction (FEC) mechanism [35]. The unlicensed sub-GHz ISM radio band is where LoRa operates. In Europe, this is between 863 and 870 MHz, whereas in the U.S., it is at 902 MHz and 928 MHz. It also abides by duty cycle constraints, which are as low as 1% in certain locations around the world. LoRa’s long-range transmission and low power consumption are particularly impressive when compared to those of short-range wireless protocols and cellular technologies. With a range of up to 15 km in rural regions and 5 km in urban areas, a battery life of up to 10 years per device, and a data rate of 0.3 to 37.5 kilobits per second [36], it is a strong contender in the wireless communications market. Compared to other wireless signals, LoRa is less likely to experience fading and more resistant to interference and intra-interference, to the Doppler effect [37]. Its devices are inexpensive, and gateways can receive many signals at once. LoRa networks in the real world seldom achieve optimal performance due to implementation complexity and varied interference.

**Figure 2:**
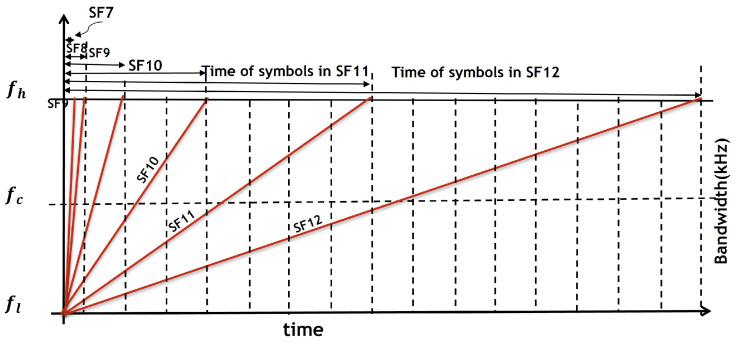
Timing of LoRa spreading factor.

The LoRa physical layer utilises the CSS modulation technique. In this technique, the data symbols are distributed using chirps, which are linear frequency-modulated sinusoidal pulses. Each chirp has a fixed bandwidth (b) defined as $b=f_{h}-f_{l}$ and a chirp time ( $T_{c}$ ) as illustrated in Figure 2. The creation of virtual channels can be achieved by manipulating the chirp duration of quasiorthogonal signals. Furthermore, the duration of the chirp introduces a trade-off between the system’s throughput and its ability to withstand noise and interference. The data symbols are encoded using a distinct instantaneous frequency trajectory, which is achieved by cyclically moving a reference chirp while keeping $T_{c}$ constant. The symbols, which are represented by cyclic shifts, are discretised into multiples of chip-time $T_{c}=1/b$ .

Additionally, there are only $2^{f}$ possible edges in the instantaneous frequency, where *f* refers to the spreading factor.

LoRaWAN nodes establish communication with gateways through the utilisation of LoRa technology. The technology in question is founded upon a CSS scheme, which is a modified version of chirp-spectrum modulation. The use of CSS, which was first developed for radar systems, has become prevalent in military and secure communication applications. This is mostly because of its ability to operate effectively with low transmission power and its inherent resilience against many channel degradation effects, including multipath, fading, Doppler, and in-band jamming interferers [11].

The Chirp Spread Spectrum technology serves as the foundational physical layer used by LoRaWAN, which is the top network stack developed by the LoRaWAN Alliance. The CSS approach is capable of accommodating various data rates. In the case of a low data rate, a significant spreading factor is used, resulting in a substantial inclusion of redundancy and energy within the sent signal. Hence, the signal can traverse considerable distances while maintaining sufficient amplitude to ensure effective reception. The aforementioned rationale applies to a sent signal with a high level of *TP*. From a radio resource management perspective, the use of high spreading factor values results in prolonged occupation of the radio medium as a consequence of reduced data rates. Hence, it is preferable to use the minimum feasible signal frequency that establishes a strong connection between the network and end devices. The use of diverse orthogonal spreading factors facilitates the achievement of numerous effective receptions by enabling the transmission of packets that overlap in both temporal and frequency domains. Despite LoRa’s limitations in the physical layer, some researchers have attempted to obtain solutions through the Medium Access Control Layer (MAC), which is named LoRaWAN and adopted by the LoRa Alliance.

The LoRaWAN specification encompasses the upper layer protocols and network architecture necessary for enabling end devices to establish direct connections with gateways. This is achieved via the use of an ALOHA-based multiple access scheme operating within the sub-GHz ISM bands. Then the gateways are linked to the NS, which carries out crucial network-level operations such as downlink transmission scheduling, the implementation of a portion of the ADR algorithm, and device authentication, among others. The LoRaWAN mechanisms adhere to the regional regulations for the use of the sub-GHz ISM band. These regulations include several aspects, including the maximum transmission power and duty cycles. This article postulates the utilisation of LoRaWAN technology inside the European region, where the duty cycle and transmission power are limited to 1% and 14 dBm, respectively, specifically for the default frequency channels. The classification of device classes is determined by the specific application requirements pertaining to both downlink communication latency and energy efficiency. The EDs with the highest energy efficiency, sometimes powered by batteries, are classified as Class A devices. These devices exhibit the greatest delay in receiving downlink messages, which are sent by the network immediately after an uplink transmission. The inclusion of different device classes offers supplementary possibilities for the reception of downlink signals, but at the cost of increased energy consumption.

### 2.2. LoRaWAN Multi-Gateway

Gateways receive LoRaWAN packets sent wirelessly from an end device and then send those packets to the network server. In addition, they transmit data packets from the network server back to the device. One important feature of these gateways is their ability to convert the received signals into binary data, which is then stored in a buffer called a packet. This packet is then sent over the gateway’s backhaul, which is the gateway’s link to the internet via Wi-Fi, cellular connection, or Ethernet. The gateways receive messages from an end device, known as uplinks, along with the accompanying information for each uplink. This metadata includes Signal-to-Noise Ratio (*SNR*), the RSSI, Frequency channel, time-of-arrival (TOA), and Data Rate. After receiving an uplink message and its corresponding metadata, the gateway transmits the message to the network server.

**Figure 3:**
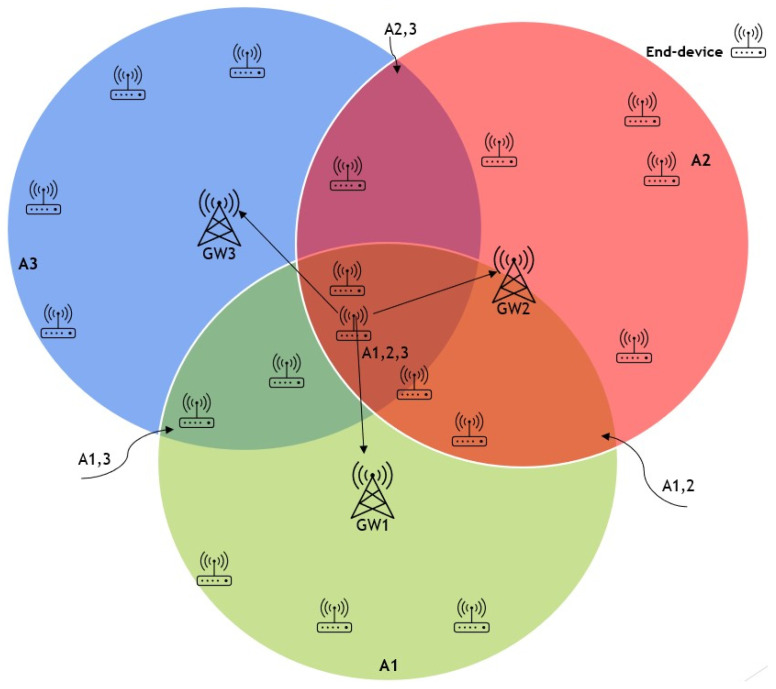
Overlapping in LoRa network deployment multi-gateways.

**Figure 4:**
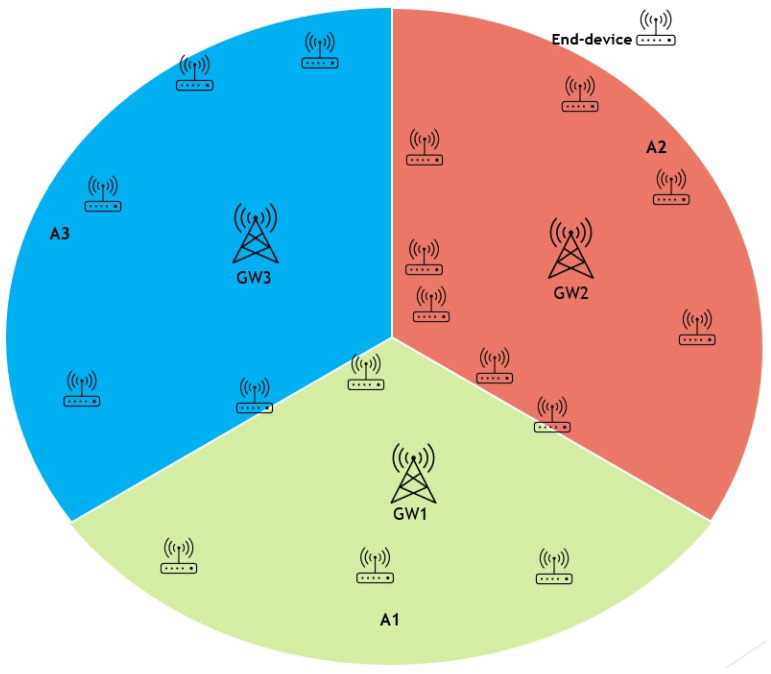
No overlapping in the proposed deployment of multiple gateways.

An advantage of implementing a LoRaWAN network is the ability to easily include extra gateways in areas where network congestion may occur due to an overload of uplink traffic, which may lead to a shortage of accessible frequencies for transmission. In some circumstances, it could be necessary to include gateways to address limitations in the available time-on-air of the gateway or to overcome network coverage issues that prevent reliable reception from end devices. Nevertheless, the extensive communication range of LoRaWAN might result in overlapping coverage areas for gateways. Consequently, packet transmissions from nodes inside these overlapping areas may clash, leading to a decline in network performance as illustrated in Figure 3. Optimising the configuration parameter values to minimise collisions and maximise network performance in multi-gateway LoRaWAN remains an unresolved issue. To address this problem, this research proposed ZBMG-LoRa to make the network adapt to the additional gateways automatically by using a low-complexity model for multi-gateway LoRaWAN. This model categorises nodes into various groups depending on the gateways they can interact with such as shows in Figure 4. Adjacent devices to new gateways will receive automated instructions to transfer data at a greater rate, reducing the time-on-air, and will be directed to transmit at a lower power level. Additionally, this will substantially enhance the longevity of the battery in battery-operated devices and enable a greater number of devices to connect to the network. This straightforward method provides a significant benefit compared to conventional mobile networks. Integrating a new base station into a conventional mobile network often requires a complete overhaul of the radio configuration, which involves modifying the frequencies used by all neighbouring base stations.

## 3. LoRaWAN Network System

When it comes to network systems, the Bit Rate (BR) is of utmost importance since it dictates how fast data can be transported. Furthermore, it guarantees that application requirements are satisfied by having an immediate effect on network performance. The Bit Rate of a LoRa network can be determined by using Equation (1) [38].

(1) $$R_{b}\;=\;f\;\times \;\frac{b}{2^{f}}\;\times \;cr\;[\mathrm{bits}\;/s]$$

cr is the coding rate, *b* is the channel bandwidth, and *f* is the spreading factor. The communication range and data rate are directly affected by the spreading factor, which in LoRa modulation (CSS) is related to the number of chips used to encode each signal. Both the susceptibility to noise and the time of each symbol transmission are determined by *f*. The spectrum of spreading factors in the context of SF7 to SF12 is characterised by a doubling in symbol duration with each incremental increase in the spreading factor, resulting in a halving of the data rate. The concept of a high spreading factor refers to the utilisation of a larger number of chips for data in a spread spectrum communication system. A spreading factor with a high value, such as SF12, offers enhanced signal resilience and facilitates long-distance communication. This technology is appropriate for situations that necessitate extensive coverage over great distances, even with the reduced data transmission rates trade-off. A low spreading factor, exemplified by SF7, is associated with increased data rates but reduced transmission range in comparison to greater spreading factors. Applications that emphasise better data throughput over long-range coverage can benefit from its usefulness. The determination of the suitable spreading factor is contingent upon the particular demands of the application, taking into account several elements such as the intended data rate, range, power consumption, and susceptibility to interference. On the contrary, the term “bandwidth” pertains to the spectrum of frequencies employed for signal transmission. The data rate in LoRa communication is directly influenced by the bandwidth, as indicated by Equation (1). The available bandwidth options are commonly set at 125 kHz, 250 kHz, or 500 kHz. A narrower bandwidth, such as 125 kHz, facilitates longer symbol lengths, hence enhancing the receiver’s sensitivity to weak signals and improving its capacity to discern varying signal levels. In order to know the range of the signal and the possibility of decoding that signal, evaluating the link budget of the signal between the gateway and end device is more significant.

### 3.1. Link Budget Model

A wireless system’s or network’s link budget is an all-inclusive evaluation of the transmission process’s gains and losses, taking into account the intended receiver, the propagation channel, and the transmitter. System gains and losses associated with the antenna, matching networks, and other parts of the system, as well as propagation channel losses that can be calculated by modelling or observational data, are all part of the gains and losses. It is common practice to provide additional margin depending on the anticipated intensity when discussing channel mechanisms that display random changes, including multipath and Doppler fading. The following is a mathematical representation of the link budget for a wireless network connection using Equation (2):

(2) $$P_{rx}\left(dBm\right)\;=\;P_{tx}\left(dBm\right)\;+\;G\left(dB\right)\;+\;PL\left(dB\right)$$

where $P_{rx}$ is the power of reciever, the $P_{tx}$ is the power of the transmitter, G(dB) is the gain of antenna for TX and RX, and the PL(dB) is the path loss for details; the summation of gain can be expressed as in Equation (3):

(3) $$G\left(dB\right)\;=\;G_{tx}\left(dB\right)\;+\;G_{rx}\left(dB\right)$$

while the all-path loss is represented in Equation (4):

(4) $$PL_{Total}\left(dB\right)\;=\;PL_{Env}\;+\;PL_{tx}\left(dB\right)\;+\;PL_{rx}\left(db\right)\;-\;X_{\sigma }$$

$G_{tx}\left(dB\right),\;$ $PL_{tx}\left(dB\right),\;$ $G_{rx}(dB)\;,$ and $PL_{rx}\left(dB\right)$ The value of plus minus is assigned to zero. The communication environment determines $PL_{Env}\left(dB\right)$ , while $X_{\sigma }$ represents the fading margin. Route loss is influenced by a variety of environments, including urban and suburban areas, in numerous models.

The popular log-distance path loss model [39] is employed to model the deployments in heavily populated areas. This architecture has been chosen because it suits LoRa deployment scenarios. This model describes path loss as a function of communication distance d as in Equation (5):

(5) $$PL_{Env}\left(d\right)\;=\;PL_{Env}\left(d_{0}\right)\;+\;10_{\lambda }log_{10}\left(\frac{d}{d_{0}}\right)\;+\;X_{\sigma }$$

$PL_{Env}\left(d\right)$ represents the path loss in *dB*, $PL_{Env}\left(d_{0}\right)$ signifies the average path loss at the reference distance $d_{0}$ , λ denotes the path-loss exponent, and $X_{\sigma }\;N\left(0,\sigma^{2}\right)$ represents the normal distribution adjusted for shadowing with a mean and a variance of zero. Additionally, the simulations conducted using the settings documented by Martin Bor [40], Mariusz Slabicki [41], Petajajarviand [42] demonstrated comparable outcomes in relation to scalability.

### 3.2. Simulation Model

Whether a receiver is able to decode one, two, or no packets at all when two LoRa signals collide depends on a number of parameters. Among these considerations are the following: power, time, carrier frequency, and spreading factor. For packets $p_{1}$ and $p_{2}$ to collide, each of the requirements specified in Equation (6) needs to be satisfied:

(6) $$C_{pckt}\;\left(p_{1},p_{2}\right)\;=\;\left\{\begin{array}{c} 1\;if\;\;(O\left(p_{1},p_{2}\right)\;\wedge \;C_{fr}\left(p_{1},p_{2}\right)\;\wedge \;C_{f}\left(p_{1},p_{2}\right)\;\wedge \;C_{pw}\left(p_{1},p_{2}\right)\;\wedge \;C_{t}\left(p_{1},p_{2}\right))\;\\ 0\;\;\;\;\mathrm{else} \end{array}\right.$$

In the case where two transmissions collide on $C_{fr}\left(p_{1},p_{2}\right)$ , it may be defined via the centre frequencies of transmission $\left(p_{1},p_{2}\right)$ , denoted as $fr_{1}$ and $fr_{2}$ , respectively. Additionally, the least allowable frequency offset is represented by a threshold. Semtech SX1272 has a minimum acceptable frequency deviation of 60 kHz when using a bandwidth of 125 kHz, 120 kHz when using a bandwidth of 250 kHz, and 240 kHz when using a bandwidth of 500 kHz. The orthogonal spreading factors $C_{f}\left(p_{1},p_{2}\right)$ are employed in this case. Therefore, it is possible to correctly decode transmissions that have different *SF* (while maintaining the same *CF* and BW), provided that there are two accessible receiver pathways. $C_{pw}\left(p_{1},p_{2}\right)$ occurs when two signals are present at the receiver, where the stronger signal suppresses the weaker signal. Thus, the received signal intensity may vary by a small degree. However, when the difference is too slight, the receiver switches between the two signals and becomes unable to decipher either, where the two packets are denoted $\left(p_{1},p_{2}\right)$ . The expression $O(p_{1},p_{2})$ represents the time complexity of a function or algorithm in terms of two variables $\left(p_{1},p_{2}\right)$ when the periods of their reception overlap. For the receiver to recognise the preamble and synchronise, it requires five symbols. Eight preamble symbols were included in the broadcasts. Hence, the receiver looks at the weak transmission after three symbols, but the strong transmission suppresses its signal, corrupting the packet. It may be inferred that packets can overlap if, in the event of a weak packet, at least five preamble symbols remain undamaged (i.e., the most important part of a packet’s receipt begins with the final five preamble symbols). Packet structure and transmission time explained in Equations (7)–(9) show how the time preamble, the symbol time, and the number or length of preamble are calculated, respectively.

(7) $$T_{\mathrm{preamble}\;}\left(f\right)\;=\;\left(L_{\mathrm{preamble}\;}\;+\;4.25\right)\;\cdot \;T_{\mathrm{symbol}\;}\left(f\right)$$

(8) $$T_{\mathrm{symbol}}\left(f\right)\;=\;\frac{2^{f}}{b}$$

(9) $$\begin{array}{c} \begin{array}{cc} L_{\mathrm{preamble}}\;=\;\left(\frac{2^{f}\times 12.25}{b}\right) & \;+\;\left\{8\;+\;\max\left(\mathrm{ceil}\left(\frac{8PL\;-\;4f\;+\;28\;+\;16CRC\;-\;20IH}{4(f\;-\;2DE)}\right)\left(CR\;+\;4\right),0\right)\right\} \end{array} \end{array}$$

Given the dependencies provided, the variable *PL* represents the quantity of payload bytes. *b* and *f* represent bandwidth and spreading factor, respectively. The value of *H* is 0 when the header is enabled and 1 when no header exists. When low data rate optimisation is enabled, the value of *DE* is 1. Conversely, when it is removed, the value of *DE* is 0. The coding rate ranges from 1 to 4. It may be inferred that if there is a need to decrease the duration of airtime and the length of the packet is predetermined, then the header data can be omitted. The duration of the payload can be calculated by multiplying the total number of payload symbols by the symbol period.

The sensitivity of the gateway and end device receivers for a given spreading factor can be denoted as $S_{G}\left(i\right)$ and $S_{e}\left(i\right)$ in decibels (dB), respectively. In [43], observation reveals that augmentation of *SF* results in improved sensitivity, with consistent increments of 2.8 dB, while decreasing BW from wider bandwidth to narrow bandwidth results in improved sensitivity, from 3 to 4 dB. For downlink (DL) transmissions, the consideration of the sensitivity of an end device is expected to be lower than that of a gateway by introducing a 3 dB offset. Sensitivity values are utilised to ascertain whether a packet is detected by a device. The sensitivities are represented by Equation (10) given in [43,44].

(10) $$S_{\left(f,b\right)}\;=\;-174\;+\;10log_{10}b\;+\;NF\;+\;SNR_{f}$$

The first term is a result of thermal noise within a bandwidth of 1 Hz and can only be altered by modifying the temperature of the receiver. The second term, *b*, refers to the bandwidth of the receiver. The receiver noise figure (*NF*) is a constant value that remains unchanged for a specific hardware implementation. The term *SNR* represents the signal-to-noise ratio that is necessary for the underlying modulation technique.

(11) $$P_{rx}\left(n\right)>S_{g}$$

If the power of a signal with a spreading factor *f* of node i at the receiver’s location falls below the threshold $S_{g}$ , it cannot be detected by the gateway. Conversely, it can be detected if the received power exceeds the necessary sensitivity. In this scenario, we also presume that the recipient will synchronise with the incoming signal and commence the reception of the packet. This suggests that once a packet is received with sufficient power to initiate detection, it will remain detectable (i.e., over the sensitivity threshold) until it is fully received. If multiple signals with individual powers below the sensitivity threshold arrive simultaneously at the receiving antenna, they cannot be recognised by the receiver, even if their combined power is above the sensitivity threshold and a collision might have happened between packets.

## 4. Zone-Based Multi-Gateway System

### 4.1. Adaptive Data Rate

The adaptive data rate (ADR) is a functionality present in the LoRaWAN protocol that allows for the automatic modification of transmission parameters specific to each end device, taking into account the current network conditions. The primary objective of ADR is to maximise network efficiency, improve capacity, and extend device battery life. ADR functions at the network server level and utilises diverse parameters to ascertain the most favourable transmission configurations for individual devices. The metrics encompass many signal quality measures, including signal-to-noise ratio, received signal intensity, and levels of network congestion. ADR can change the essential transmission parameters, including the *SF*, CR, and transmission power (*TP*). The spreading factor is a crucial parameter that influences the bandwidth of the signal and has a significant impact on both the transmission range and data throughput. Increasing the spreading factor results in an extended coverage area, hence enhancing the range of communication. However, this comes at the cost of reduced data transmission rates. ADR is a mechanism that modifies the spreading factor in response to the prevailing signal conditions, with the aim of achieving an optimal trade-off between the data rate and the range of communication [45]. The objective of the standard ADR algorithms is to coordinate the value of *SF* and *TP*, hence facilitating connectivity between the ^GW^ and the ED, with the aim of minimising energy consumption. The method implemented on the network server is developed by the server developer, whereas the algorithm operating on the ED is specified by the LoRa Alliance [8]. The NS algorithm commonly refers to the implementation utilised by prominent platforms such as The Things Network or The Stack Things. This implementation is founded on Semtech’s suggested method [46]. ADR in LoRaWAN facilitates the adaptive modification of transmission parameters between a node and the network server. When a node tries to become part of the network, it utilises the device’s default transmission parameters for connectivity and sets the ADR counter to zero. The counter is incremented with each subsequent transmission, and it resets to 0 upon the reception of an ACK. Following a series of transmissions that did not receive a response more than (ADR_ACK _LIMIT), the value of Pt is augmented. When the node achieves its maximum *TP*, it subsequently increases the *SF* to become connected with the NS. The aforementioned procedure is iterated for each ADR_ACK_DELAY transmission until either a response is successfully received or the node achieves the maximum values of spreading factor *f* and transimission power. The customizable settings of ADR_ACK _LIMIT and ADR_ACK_DELAY are set to a default value of 32 [41]. The network server assesses the quality of the connection. The evaluation of connection quality by the NS is accomplished by utilising signal-to-noise ratio measurements conducted at the *GWs*. The method calculates the necessary number of Pt or *SF* adjustment steps to achieve a stable communication state, considering factors such as link quality, sensitivity for each spreading factor and SNR [47], and an error margin (margin_db). The channel estimate process takes into account the highest *SNR* value, denoted as SNR−m , obtained from the N most recent transmissions. The value of the variable $N_{step}$ is equal to the number of changes in connection strength. The node modifies its settings in response and carries out further communications. This repeated reconfiguration leads to more consuming power of nodes. Another concern with this strategy is the magnitude of the step in $P_{t}$ level and *SF*. Some researchers have attempted to address this issue by reducing the increase in $TP_{level}$ by 1 dBm, as demonstrated in [41,48,49]. However, the step size of *SF* remains unchanged. In our proposed approach, we recommend employing SDR to reduce this step size.

### 4.2. ZBMG-LoRa System

We assume that a set of N nodes exists that are randomly distributed across the coverage area of 7000 m^2^, with four gateways, each with three main channels. Our solution has two steps: firstly, to avoid the behaviour of LoRa sending to all gateways when the network infrastructure contains multiple gateways. Although this is an advantage of redundancy in terms of highly guaranteeing packet reception, it leads to high collisions and large energy consumption. The optimal setting for the node could guarantee a successful reception of the packet. Disabling multicast addressing and enabling unicast addressing should be returned with F-setting to activate the unicast DevAddr for the node. Our study aims to quantify the mean likelihood of achieving successful packet reception for each node in every gateway zone in the network.

Accordingly, the proportion of nodes configured with a gateway *g* is calculated using Equation (12).

(12) $$\sum_{i=1}^{G}\alpha_{g}=1\;\forall g\in GWs$$

where *g* from 1 to G refers to the maximum number of gateways. The LoRa MAC layer is based on ALOHA MAC protocol that operates without acknowledgements. It is assumed that the nodes transmit packets autonomously, without any dependence on each other or their geographical locations. The proportion of nodes configured with a spreading factor *f* is given in [45].

(13) $$\sum_{i=7}^{12}\zeta_{f}=1\;\forall f\in SFs$$

**Figure 5:**
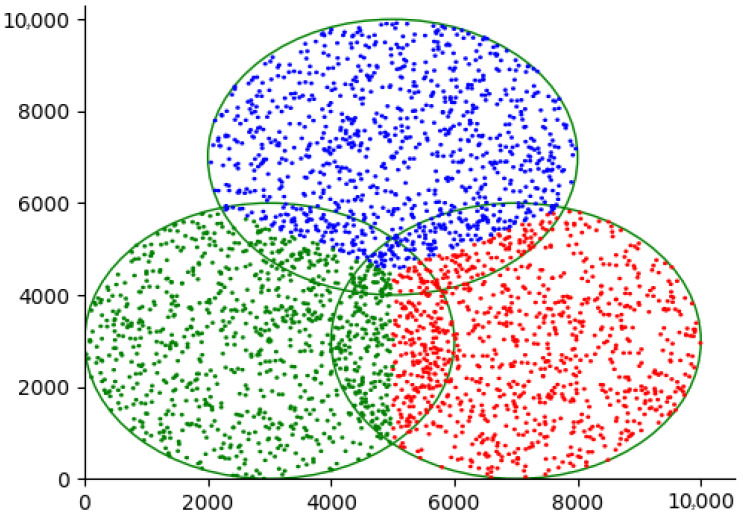
No overlapping in ZBMG-LoRa area (m^2^) from the simulator.

**Figure 6:**
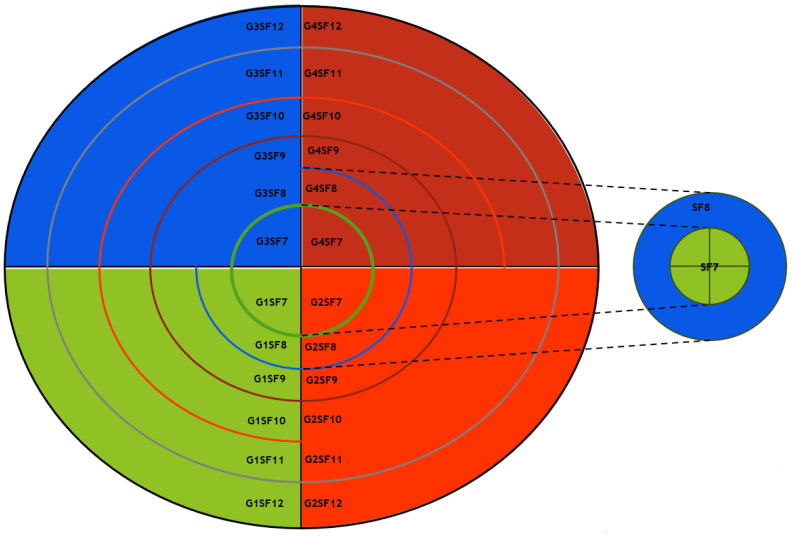
ZBMG-LoRa deployment strategy.

**Figure 7:**
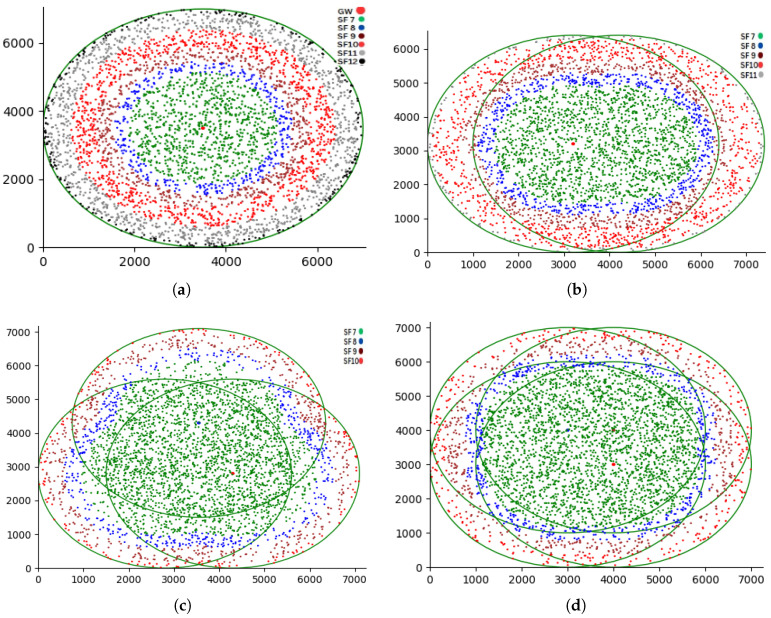
(**a**) One gateway scenario. (**b**) Two gateway scenario. (**c**) Three gateway scenario. (**d**) Four gateway scenario.

For all values *f* between 7 and 12. Figure 5 and Figure 6 depicts the deployment of gateway-based and spreading factor-based strategies. respectively. Figure 7a shows that the Poisson distribution for packet generation at the nodes’ deployment area is $G_{f}$ across all gateway zones and $zone_{g,f}$ . Here, we pretend a node di away from the gateway is sending μ messages with a *f* spreading factor. By analysing the signal’s path-loss characteristics, as shown in Equation (5), we may identify possible sources of interference, assuming that all nodes have specific transmission parameters. The maximum distance between any node and the gateway is specified by Equation (14).

(14) $$D=d_{0}\times 10^{\frac{PL_{env}}{10\lambda }}$$

where λ represents the path-loss exponent. Therefore, by assuming a random distribution of nodes with a spreading factor *f*, as a single gateway scenario [50], our research discussed multiple gateways *g* scenario. So, the total number of possible interferers may be calculated. $\alpha_{g}N\frac{(min{\left(d_{i},D\right)}^{2}}{D^{2}}$ where *D* is the range. $P_{s}\left(d\right)$ is the probability of successful transmission. To maintain a reliable and secure system, it is essential to guarantee that no potential interfering nodes initiate a transmission during a vulnerability period of 2T*f*.

(15) $$P_{s}\left(d\right)=e^{-2T_{f}\mu \alpha_{g}N\left(\frac{{\left(min\left(d_{i},D\right)\right)}^{2}}{D^{2}}\right)}$$

Given the capture effect, there are two ways to tell if a node has successfully transmitted a packet: (a) No other packet with the same spreading factor has overlapped with it within the same receiving time $t_{f}$ , or (b) the power level at the gateway of any other packet with the same *SF* is higher than the current packet’s power level by at least a certain threshold value $PW_{thld}$ , as shown in Equation (6).

The data rate probability is derived from Equation (1). Meanwhile, the likelihood of success data rate is derived from the equation introduced in [45].

(16) $$Pc_{f}=\frac{f}{2^{f}}/\sum_{i=7}^{12}\frac{i}{2^{i}}\;\forall f\in SFs$$

However, neither Equation (16) nor (12) takes into account the *SF*, and neither does Equation (16) take into account the gate amount *g* or the channel variables. It is critical to consider the impact of two key elements—channels and spreading factors—in order to precisely forecast the likelihood of our approach’s success. We can optimise our technique for optimum efficiency and effectiveness by considering the effects of *GW*, *SF*, and *Ch* on the chance of success. The throughput Tht and success probability Equation (15) can be determined using Equations (12) and (17).

(17) $$P_{s}\left(d\right)=e^{-2T_{f}\mu \zeta_{f}\alpha_{g}\left(N\frac{{\left(min\left(d_{i},D\right)\right)}^{2}}{D^{2}}\right)}$$

(18) $$Tht=\frac{P_{s}\times P_{L}\times N}{\tau }$$

where $P_{L}$ is the data packet and *N* is the number of nodes. $P_{g,f}$ is the collision probability expressed by Equation (19).

(19) $$P_{g,f}=\frac{p_{g}*f}{\sum_{i\in GWs}i}\;\forall g\in GW\&sf\in SFs$$

Moreover, considering multiple channel frequencies, Equation (19) becomes the following:

(20) $$P_{f,b,ch}=\frac{p_{g,f}*ch}{\sum_{i\in CHs}i}\forall g\in GWs\&f\in SFs\&ch\in CHs,$$

Our algorithm illustrates the operation of the technique, which involves the allocation of network nodes according to the proximity of the gateway and the minimum path loss. The following step is to divide that gateway area into tiny zones for each spreading factor; for instance, we have four gateways in the network field. The whole area is divided into four zones G1 through G4. Then, each zone area divided into six *f* zones is delineated according to the spreading factors, as depicted in Figure 6. So, the total small zones will be 24 Z, and all nodes belonging to those zones will be allocated to different channels according to Equation (20).

### 4.3. ZBMG-LoRa Algorithm

The ZBMG-LoRa algorithm is proposed in this section. The initial step in establishing a connection between the nodes and the gateway involves utilising default settings, which entail a high spreading factor, narrow bandwidth, and a small code rate. During this connection process, the gateway receives a substantial amount of information from the nodes, including *SNR*, RSSI, and various other settings, which are transferred to the network server. Firstly, the network server defines transmission power levels as shown in the ZBMG-LoRa-LoRa Algorithm 1 $P_{tx}level$ , SF , *B*, and Ch , then lists the settings depending on the number of *GWs*, as illustrated in Pseudocode row 1. Secondly, the NS sorts the nodes and gateway in the matrix depending on PLoss , not *d* distance. Subsequently, as illustrated from row 3 to row 7, the algorithm proceeds to evaluate the connection and link budget by comparing the nodes’ RSSI values to the gateway’s sensitivity. Based on the node’s link budget and its RSSI, the best setting will be selected from multiple configurations Set[f,B,Cr,g,TP] , which returns the best gateway for a reliable connection. The main objective is to select the optimal setting that ensures a reliable connection while minimising power consumption. In our approach, the selection of node configuration is not solely based on transmission power and spreading factor as in conventional methods. Instead, the multi-data rate is incorporated, which takes into account both the multi-gateway, spreading factor and multi-channel, in addition to the slim *TP* steps by 1 dBm. The ZBMG-LoRa algorithm is designed based on our previous work MBMD-LoRa [44] to determine the optimal route and rank for a novel technique, with the objective of establishing an efficient and reliable connection. The network server initiates the assessment of the RSSI of nodes and compares it with the gateways’ sensitivity. It then assigns them a configuration which aligns with the specific circumstances of each node and assigns that node to the proposed gateway by increasing the value of *k* in zone gf . If the node’s RSSI is not more than the gateway sensitivity, the algorithm increases the transmission power of that node, as in rows 25 to 29, then reassessment of the node’s link budget occurs as in row 6 to obtain a suitable setting $set_{i}$ for the node, and so on. The use of diverse transmission characteristics, such as spreading factors and gateways, leads to different settings zones and more available channels. As a result, the disparity in airtime allocation results in differing collision probabilities, hence creating an unfair distribution of resources among nodes inside a certain zone. The ZBMG-LoRa algorithm is proposed using ζf and αg to ensure fair distribution and enhance the packet delivery ratio based on ζ which is the result of the multiplication of Equations (12) and (13), which increase the number of settings. As noted, the algorithm is checking if the number of nodes in Zone $Z_{f}$ assigned to spreading factor *f* is not more than the $\zeta_{f}$ value; otherwise, a move to fulfil the next zone $Z_{f+1}$ is made, and so on, based on the following equations:

(21) $$\sum_{i=7}^{12}Zone_{f}=1\;\forall f\in SFs$$

(22) $$\sum_{j=1}^{G}\alpha_{b}\sum_{i=7}^{12}\zeta_{f}=1\;\forall f\in SFs,\forall g\in GWs$$

Our strategic initiative, ZBMG-LoRa, seeks to enhance LoRaWAN, whereby the doubling of packet reception is not designed to decrease packet transmission time, resulting in fewer collisions and less transmission power. The level of variability in *SF* inside an individual cell substantially affects Pckts ’ capacity to evade collisions.

Our approach will enhance energy efficiency, primarily because an increased data rate leads to a reduction in Pckts time, coupled with a drop in collisions attributable to Pckt size, hence diminishing transmission power and the need for Pckt retransmission. If the node moves to a different location, its setting will be reevaluated to obtain a new setting that is suitable for the new location.
**Algorithm 1:** ZBMG-LoRa Algorithm

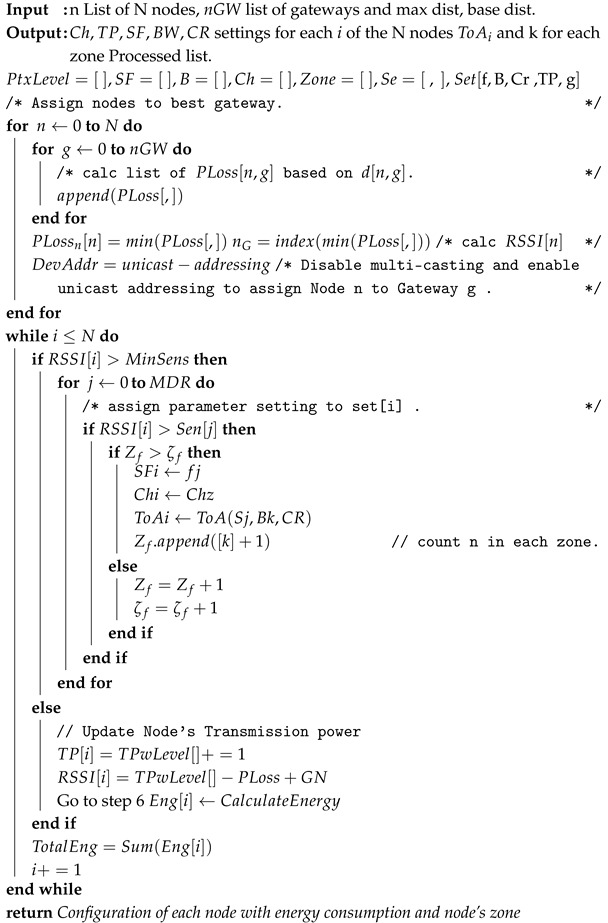



## 5. Performance Evaluation

This section demonstrates the efficacy of our suggested resource allocation strategies, which use the zone-based multi-gateway ZBMG-LoRa algorithm. Our prior research indicates that increasing the number of base stations is a method to enhance the scalability of LoRa networks [44]. This section reports on tests to assess the validity of this assertion in ZBMG-LoRa configurations. We substitute the base station in the middle of the configuration shown in Figure 7 with two or three base stations, respectively. We position these supplementary base stations at a distance d from the primary base station. For the two-station scenario, we shift the original base station d to the right while maintaining its vertical position and introduce a second base station d to the left of the original site. When substituting the original base station with three base stations, we elevate one base station by distance d while the other two are positioned at 45 degrees below and laterally to the left and right, respectively, maintaining a distance of d from the original base station’s location. The positioning of the sensor nodes remains constant; they are situated within a radius *r* around the base station’s initial location. A packet transmission is deemed successful if it is received by any of the base stations. All four interfering networks are operational. Their performance is assessed using a model derived from LoRaSim, a discrete-event simulator created by Bor et al. [40]. Additionally, the Simpy library is used to examine collision and scalability challenges in LoRa networks. Simulations are constructed using Python 3.9. A distinct approach has been created that mainly varies in the allocation of *SF*s, where specifically the ZBMG-LoRa-LoRa is a gateway-based allocation with $\alpha_{g}$ six distributed zones $\zeta_{f}$ . Given that our approaches are designed for large-scale dense networks, the simulations were conducted with a substantial number of nodes, ranging from 500 to 4000.

These nodes were randomly dispersed throughout a 7 km^2^ geographical area. In addition, we assumed the presence of a LoRa network with one to four gateways positioned at the centre of the region with a constant distance between them. In terms of packet size, each node produces packets that are 40 bytes in size. The duration between the arrival of consecutive packets follows an exponential distribution with a mean of 200 s. Similar to the study in [49], the European regional specifications for the LoRa physical layer are employed, with a 1% duty cycle for both the LoRa nodes and the gateway. Table 1 presents a summary of the simulation parameters that were utilised. We conduct an analysis and comparison of the effectiveness of our approach with LoRaWAN which included ADR. This evaluation is based on the assessment of collision probability, throughput, and packet delivery ratio (PDR).

Our proposed methodology, referred to as the ZBMG-LoRa framework, aims to improve and optimise the performance of LoRaWAN technology. Figure 7 show the zone of spreading factor 7 became wider due to the distance between the gateways. Spreading factor 7 has the shortest time-on-air and the highest data rate, and it is also the most power efficient. On the other hand, the absence of spreading factors 11 and 12 enhances the fairness between the nodes. Our proposed methodology achieves higher energy efficiency. This could be attributed mainly to two factors. Firstly, the increase in data rate results in a decrease in the packet transmission time (ToA). Secondly, reduced collision occurrence owing to packet size leads to lower transmission power and fewer packet retransmissions. Additionally, cellular network channel diversity prevents node collisions, hence saving energy by avoiding a packet resend.

The algorithm underwent evaluation through comparison with both the standard solution in the field and an alternative approach described in [13,40]. This evaluation employs an integrated methodology that encompasses both simulations and tests with two scenarios. The first study examines the impact of an increased number of gateways on our approach, while the second compares our approach to traditional studies in different performance metrics such as collisions, packet delivery ratio, energy consumption, and network throughput. The low complexity in our optimisation problem does not depend on the number of nodes. In our proposed ZBMG-LoRa, we find the optimal node distribution for each *SF* by computing their RSSI with a low computational time.

### 5.1. Packet Delivery Ratio (PDR)

**Figure 8:**
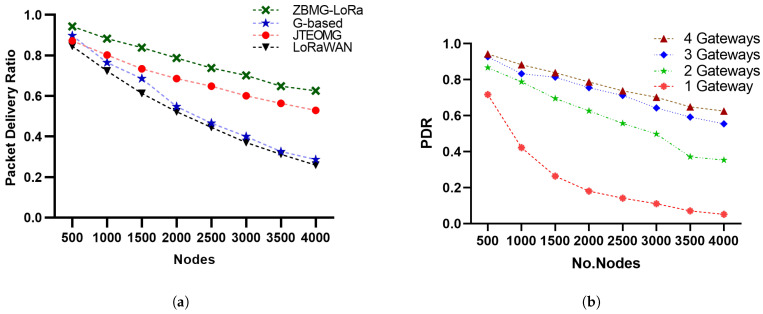
(**a**): Packet delivery ratio of Methods, (**b**): Packet delivery ratio with multiple gateways.

**Figure 9:**
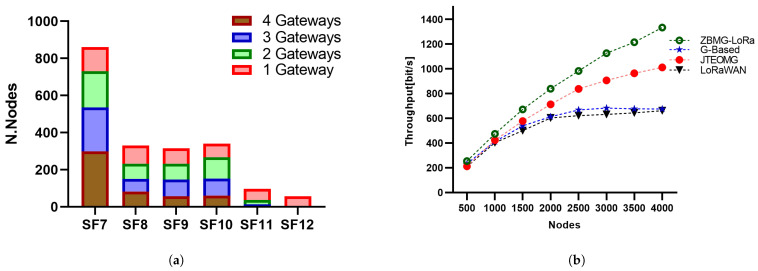
(**a**): Spreading factor distribution, (**b**): Throughput as a function of nodes.

The Packet Delivery Ratio is the quotient of the total number of received packets higher than the gateway sensitivity. According to Figure 8a, the PDR of the ZBMG-LoRa scenario in the proposed approach is much higher than the PDR of joint throughput-energy optimisation in multi-gateway LoRaWAN networks JTEOMG [13] and LoRaWAN. More precisely, the PDR of our approach is three times higher than the PDR of LoRaWAN when there are 4000 nodes. In multi-gateway scenario there is a significant improvement when using more gateways according number of gateways as illustrated in Figure 8b. As a result of extending the lower spreading factors zones between gateways most of nodes grant SF7 that why achieve higher data rate and short ToA of packets as illustrated in Figure 9a. The difference is minimal with four gateways, indicating that three gateways can provide effective coverage in the area.

### 5.2. Throughput

Figure 9b illustrates the network throughput as a variable dependent on the number of EDs. The performance in this instance is a direct result of the conduct of the PDR. For small values of nodes, the Tht grows proportionally with increasing number of nodes, as indicated by Equation (18). This leads to a high PDR. However, when the number of nodes becomes excessively large, the PDR starts to decrease significantly, causing a saturation effect on the throughput. Hence, the enhancement of the suggested solutions in comparison to the conventional one is amplified by augmenting the quantity of EDs. To begin with, it is important to note that ZBMG-LoRa iterations of our protocol attain superior data transfer rates compared to LoRaWAN. Furthermore, it should be noted that the throughput of LoRaWAN reaches a state of stability once the number of nodes reaches 2000. Succinctly, the LoRaWAN throughput has reached its maximum capacity at this stage. In contrast, the throughput of our suggested protocol demonstrates a distinct rise in bit per second as the number of nodes increases. Therefore, our approach exhibits enhanced scalability compared to LoRaWAN. When the number of nodes, denoted as N, is set to 6000, the throughput of our proposed protocol utilising ZBMD-LoRa is more than twice as high as the throughput achieved with LoRaWAN.

### 5.3. Energy Consumption

**Figure 10:**
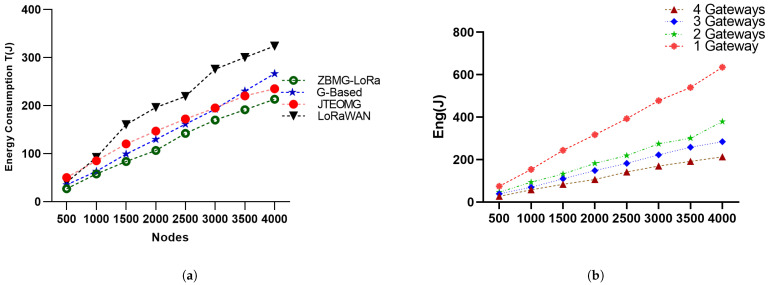
(**a**): The energy consumption of methods, (**b**): The energy consumption with respect to the number of gateways.

Figure 10a clearly presents the total energy usage per T period for each scenario. The energy consumption is accurately calculated using the total energy expended by all LoRa nodes and the number of packets sent. ZBMG-LoRa exhibits a notable reduction in energy use. These findings demonstrate the significant improvements in energy efficiency demonstrated by the proposed MBMD-LoRa algorithm performance. The energy-saving technique involves using SF7, SF8, and SF9 extensively, with minimal use of SF10, and completely avoiding SF11 and SF12 in the ZBMG-LoRa method. In contrast to the legacy versions of LoRaWAN and JTEOMG, which use all *SF* levels, our ZBMG-LoRa is more energy efficient. The random distribution in LoRaWAN fails to exploit all levels between the same spreading factors, in contrast to our approach, which effectively utilises all levels within a single spreading factor. In a multi-gateway scenario, there is a significant improvement in energy consumption when using more gateways according number of gateways. As a result of extending the lower spreading factors zones between gateways, most of the nodes grant SF7, which leads to achieving a short ToA for packets, as illustrated in Figure 10b.

## 6. Conclusions

This paper introduces a novel Zone-based Multi-gateway (ZBMG) method designed to achieve scalable communication in long-range IoT networks. The ZBMG-LoRa algorithm improves the data rate by eliminating the use of SF11 and SF12, which have the longest transmission times and contribute to channel congestion. As a result, this enhancement reduces packet transmission time and significantly improves energy efficiency. An increase in data rate leads to a reduction in packet time-of-arrival (ToA), which also decreases the number of collisions related to packet size. This reduction in number of collisions lowers the transmission power required and minimises the need for packet retransmissions.

A key feature of this method is the introduction of diverse channels within the cell, which effectively prevents collisions between nodes. This improves energy conservation by reducing the necessity for packet retransmissions. Simulation results demonstrate that our proposed approach offers a superior packet delivery ratio and lower energy consumption compared to existing methods like JTEOMG and LoRaWAN. It is important to note that a substantial number of nodes within overlapping coverage areas must optimise their settings to maximise network throughput. This highlights a clear trade-off between efficiency and fairness, an issue that will require significant attention in our future work.

## Figures and Tables

**Table 1:** Evaluation parameters [41].

Parameter	Value	Comments
N	500 → 4000	Nodes in network
GW	1 → 4	Gateways
*f*	7 → 12	Spreading factors
d0	1000 m	initial distance
λ	2.32 dBm	PLoss exponent
$PL_{Env}\left(d0\right)$	128.95	Ploss of initial distance
TPLevel	2 → 14 dBm	Transmission Power
cr	4/5	Coding Rate
*b*	125 kHz	Bandwidth
Area	7000 m^2^	Field Area
CF	[860, 864, 868]	Carrier Frequency (MHz)
T(s)	One day	Simulation time
τ	10 min	Round time

## Data Availability

The original contributions presented in this study are included in the article. Further inquiries can be directed to the corresponding author.
