# Correction: Almuhaya et al. ZBMG-LoRa: A Novel Zone-Based Multi-Gateway Approach Towards Scalable LoRaWANs for Internet of Things. *Sensors* 2025, *25*, 5457

**DOI:** 10.3390/s26061924

**Published:** 2026-03-19

**Authors:** Mukarram Almuhaya, Tawfik Al-Hadhrami, David J. Brown, Sultan Noman Qasem

**Affiliations:** 1 Computer Science Department, School of Science and Technology, Nottingham Trent University, Clifton, Nottingham NG11 8NS, UK; nmnmzair@gmail.com (M.A.); tawfik.al-hadhrami@ntu.ac.uk (T.A.-H.);; 2 Computer Science Department, College of Computer and Information Sciences, Imam Mohammad Ibn Saud Islamic University (IMSIU), Riyadh 11432, Saudi Arabia

## Missing Citation

In the original publication [1], [50] was not cited. The citation has now been inserted in Section 4.2 (ZBMG-LoRa System), Paragraph 3.

The sentence “Therefore, by assuming a random distribution of nodes with a spreading factor *f*, the total number of possible interferers may be calculated” has been replaced with “Therefore, by assuming a random distribution of nodes with a spreading factor *f*, as a single gateway scenario [50], our research discussed multiple gateways *g* scenario. So, the total number of possible interferers may be calculated.”

## Correction of References

50. Zorbas, D.; Maillé, P.; O’Flynn, B.; Douligeris, C. Fast and Reliable LoRa-based Data Transmissions. In Proceedings of the IEEE Symposium on Computers and Communications (ISCC), Barcelona, Spain, 29 June–3 July 2019. Available online: https://ieeexplore.ieee.org/document/8969766/authors (accessed on 4 March 2026).

With this correction, the order of some references has been adjusted accordingly.

The authors state that the scientific conclusions are unaffected. This correction was approved by the Academic Editor. The original publication has also been updated.
